# Evidence for *SMAD3 *as a modifier of breast cancer risk in *BRCA2 *mutation carriers

**DOI:** 10.1186/bcr2785

**Published:** 2010-11-29

**Authors:** Logan C Walker, Zachary S Fredericksen, Xianshu Wang, Robert Tarrell, Vernon S Pankratz, Noralane M Lindor, Jonathan Beesley, Sue Healey, Xiaoqing Chen, Dominique Stoppa-Lyonnet, Carole Tirapo, Sophie Giraud, Sylvie Mazoyer, Danièle Muller, Jean-Pierre Fricker, Capucine Delnatte, Rita K Schmutzler, Barbara Wappenschmidt, Christoph Engel, Ines Schönbuchner, Helmut Deissler, Alfons Meindl, Frans B Hogervorst, Martijn Verheus, Maartje J Hooning, Ans MW van den Ouweland, Marcel R Nelen, Margreet GEM Ausems, Cora M Aalfs, Christi J van Asperen, Peter Devilee, Monique M Gerrits, Quinten Waisfisz, Csilla I Szabo, Douglas F Easton, Susan Peock, Margaret Cook, Clare T Oliver, Debra Frost, Patricia Harrington, D Gareth Evans, Fiona Lalloo, Ros Eeles, Louise Izatt, Carol Chu, Rosemarie Davidson, Diana Eccles, Kai-Ren Ong, Jackie Cook, Tim Rebbeck, Katherine L Nathanson, Susan M Domchek, Christian F Singer, Daphne Gschwantler-Kaulich, Anne-Catharina Dressler, Georg Pfeiler, Andrew K Godwin, Tuomas Heikkinen, Heli Nevanlinna, Bjarni A Agnarsson, Maria Adelaide Caligo, Håkan Olsson, Ulf Kristoffersson, Annelie Liljegren, Brita Arver, Per Karlsson, Beatrice Melin, Olga M Sinilnikova, Lesley McGuffog, Antonis C Antoniou, Georgia Chenevix-Trench, Amanda B Spurdle, Fergus J Couch

**Affiliations:** 1 Division of Genetics and Population Health, Queensland Institute of Medical Research, 300 Herston Road, Brisbane 4029, Australia; 2 Department of Laboratory Medicine and Pathology, Mayo Clinic, 200 First Street SW, Rochester, MN 55905, USA; 3 Research Division, Peter MacCallum Cancer Center, A'Beckett Street, Melbourne, VIC 8006, Australia; 4 INSERM U509, Service de Génétique Oncologique, Institut Curie, Université Paris-Descartes, 26 rue d'Ulm, 75248 Paris cedex 05, France; 5 Unité Mixte de Génétique Constitutionnelle des Cancers Fréquents, Centre Hospitalier Universitaire de Lyon/Centre Léon Bérard, 28 Rue Laennec, 69008 Lyon, France; 6 Equipe labellisée LIGUE 2008, UMR5201 CNRS, Centre Léon Bérard, Université de Lyon, 28 Rue Laennec, 69008 Lyon, France; 7 Unité d'Oncogénétique, CLCC Paul Strauss, 3 rue de la Porte de l'Hoˆpital BP42, 67065 Strasbourg Cedex, France; 8 Centre René Gauducheau, Boulevard Jacques Monod, Nantes 44805 Saint Herblain Cedex, France; 9 Cancer Genetics Network 12 "Groupe Génétique et Cancer", Fédération Nationale des Centres de Lutte Contre le Cancer, 101 Rue de Tolbiac, 75654 Paris Cedex 13, France; 10 Centre for Hereditary Breast and Ovarian Cancer, Department of Obstetrics and Gynaecology, University of Cologne, Albertus-Magnus-Platz, 50923 Cologne, Germany; 11 Institute for Medical Informatics, Statistics and Epidemiology, University of Leipzig, Ritterstraße 26, 04109 Leipzig, Germany; 12 Institute of Human Genetics, University of Würzburg, Sander Ring 2, 97070 Würzburg, Germany; 13 Department of Obstetrics and Gynaecology, University of Ulm, Oberer Eselsberg 11, 89069 Ulm, Germany; 14 Department of Obstetrics and Gynaecology, Division of Tumor Genetics, Klinikum rechts der Isar, Technical University Munich, Arcisstraße 21, 80333 Munich, Germany; 15 Family Cancer Clinic, The Netherlands Cancer Institute, Plesmanlaan 121, Amsterdam 1066 CX, The Netherlands; 16 Department of Epidemiology, Netherlands Cancer Institute, Plesmanlaan 121, Amsterdam 1066 CX, The Netherlands; 17 Department of Medical Oncology, Family Cancer Clinic, Erasmus University Medical Center, Groene Hilledijk 301, 3075 EA Rotterdam, The Netherlands; 18 Department of Clinical Genetics, Family Cancer Clinic, Erasmus University Medical Center, Groene Hilledijk 301, 3075 EA Rotterdam, The Netherlands; 19 Department of Human Genetics 849, Radboud University Nijmegen Medical Centre, Geert Grooteplein Zuid 10, 6525 GA Nijmegen, The Netherlands; 20 Department of Medical Genetics, University Medical Center Utrecht, Heidelberglaan 100, Utrecht 3584 CX, The Netherlands; 21 Department of Clinical Genetics, Academic Medical Center, Meibergdreef 9, Amsterdam 1105 AZ, The Netherlands; 22 Department of Clinical Genetics, Leiden University Medical Center, Albinusdreef 2, 2333 ZA Leiden, The Netherlands; 23 Department of Human Genetics & Department of Pathology, Leiden University Medical Center, Albinusdreef 2, 2333 ZA Leiden, The Netherlands; 24 Department of Genetics and Cell Biology, University Medical Center, P. Debyelaan 25, 6229 HX Maastricht, The Netherlands; 25 Department of Clinical Genetics, VU University Medical Center, De Boelelaan 1105, 1081 HV Amsterdam, The Netherlands; 26 Centre for Cancer Genetic Epidemiology, Department of Public Health and Primary Care, University of Cambridge, Strangeways Research Laboratory, Worts Causeway, Cambridge CB1 8RN, UK; 27 Centre for Cancer Genetic Epidemiology, Department of Oncology, University of Cambridge, Strangeways Research Laboratory, Worts Causeway, Cambridge CB1 8RN, UK; 28 Genetic Medicine, Manchester Academic Health Sciences Centre, Central Manchester University Hospitals NHS Foundation Trust, St Mary's Hospital, Hathersage Road, Manchester M13 9LW, UK; 29 Oncogenetics Team, The Institute of Cancer Research and Royal Marsden NHS Foundation Trust, 15 Cotswold Road, Sutton, Surrey SM2 5NG, UK; 30 Clinical Genetics Department, Guy's and St Thomas NHS Foundation Trust, Guys Hospital, St Thomas Street, London SE1 9RT, UK; 31 Yorkshire Regional Genetics Service, St. James's Hospital, Beckett Street, Leeds LS9 TF7, UK; 32 Ferguson-Smith Centre for Clinical Genetics, Block 4 Yorhill NHS Trust, Yorkhill, Glasgow G3 8SJ, UK; 33 Wessex Clinical Genetics Service and Cancer Sciences Division, Princess Anne Hospital, Southampton SO16 5YA, UK; 34 West Midlands Regional Genetics Service, Birmingham Women's Hospital Healthcare NHS Trust, Mindelsohn Way, Edgbaston, Birmingham B15 2TG, UK; 35 Sheffield Clinical Genetics Service, Sheffield Children's Hospital, Western Bank, Sheffield, S10 2JF, UK; 36 Abramson Cancer Center, University of Pennsylvania School of Medicine, 531 BRB 2/3, 421 Curie Boulevard, Philadelphia, PA 19104, USA; 37 Division of Special Gynecology, Department of OB/GYN, Medical University of Vienna, Waehringer Guertel 18-20, 1090 Vienna, Austria; 38 Women's Cancer Program, Fox Chase Cancer Center, 333 Cottman Avenue, Philadelphia, PA 19111, USA; 39 Department of Obstetrics and Gynecology, Helsinki University Central Hospital, Haartmaninkatu 8, 00290 Helsinki, Finland; 40 Department of Pathology, University Hospital and University of Iceland School of Medicine, 101 Reykjavik, Iceland; 41 Section of Genetic Oncology, University Hospital of Pisa, Via Roma 57, Pisa 56127, Italy; 42 Department of Oncology, Lund University Hospital, S-22185 Lund, Sweden; 43 Department of Clinical Genetics, Lund University Hospital, S-22185 Lund, Sweden; 44 Department of Oncology, Karolinska University Hospital, 171 64 Solna, Stockholm, Sweden; 45 Department of Oncology, Sahlgrenska University Hospital, S-41345 Gothenburg, Sweden; 46 Department of Radiation Sciences, Oncology, Umeå University, SE-901 87 Umeå, Sweden; 47 Department of Oncology, Clinical Sciences Lund, Lund University, SE 221 85 Lund, Sweden

## Abstract

**Introduction:**

Current attempts to identify genetic modifiers of *BRCA1 *and *BRCA2 *associated risk have focused on a candidate gene approach, based on knowledge of gene functions, or the development of large genome-wide association studies. In this study, we evaluated 24 SNPs tagged to 14 candidate genes derived through a novel approach that analysed gene expression differences to prioritise candidate modifier genes for association studies.

**Methods:**

We successfully genotyped 24 SNPs in a cohort of up to 4,724 *BRCA1 *and 2,693 *BRCA2 *female mutation carriers from 15 study groups and assessed whether these variants were associated with risk of breast cancer in *BRCA1 *and *BRCA2 *mutation carriers.

**Results:**

SNPs in five of the 14 candidate genes showed evidence of association with breast cancer risk for *BRCA1 *or *BRCA2 *carriers (*P *< 0.05). Notably, the minor alleles of two SNPs (rs7166081 and rs3825977) in high linkage disequilibrium (*r*^2 ^= 0.77), located at the *SMAD3 *locus (15q22), were each associated with increased breast cancer risk for *BRCA2 *mutation carriers (relative risk = 1.25, 95% confidence interval = 1.07 to 1.45, *P*_trend _= 0.004; and relative risk = 1.20, 95% confidence interval = 1.03 to 1.40, *P*_trend _= 0.018).

**Conclusions:**

This study provides evidence that the *SMAD3 *gene, which encodes a key regulatory protein in the transforming growth factor beta signalling pathway and is known to interact directly with *BRCA2*, may contribute to increased risk of breast cancer in *BRCA2 *mutation carriers. This finding suggests that genes with expression associated with *BRCA1 *and *BRCA2 *mutation status are enriched for the presence of common genetic modifiers of breast cancer risk in these populations.

## Introduction

*BRCA1 *and *BRCA2 *mutation carriers are at increased risk for developing breast cancer and/or ovarian cancer. Estimates of the cumulative risk of breast cancer by age 70 years range from 46% to 87% for *BRCA1 *mutation carriers and from 43% to 84% for *BRCA2 *mutation carriers [1-6]. Evidence from these studies suggests that breast cancer risks in mutation carriers are modified by environmental or genetic factors. A number of large studies, facilitated through the Consortium of Investigators of Modifiers of *BRCA1*/*BRCA2 *(CIMBA), have evaluated associations between genetic polymorphisms and breast cancer risk in *BRCA1 *and *BRCA2 *mutation carriers [7-15].

The candidate gene (or candidate SNP) approach for identifying potential risk modifiers has been successfully used to identify a SNP in the 5' untranslated region of *RAD51*. Until recently, this finding has provided the most reliable evidence for a genetic modifier in *BRCA2 *mutation carriers [7]. A major disadvantage of using this approach to identify common genetic modifiers of breast cancer, however, is the limited understanding of mechanisms and pathways that underlie breast cancer development in families carrying mutations in *BRCA1 *or *BRCA2*. An alternative and powerful approach that can overcome such issues is the use of genome-wide association (GWA) studies to identify candidate SNPs. Analysis of breast cancer risk-associated SNPs identified by a large population-based GWA study of breast cancer [16] has shown that several of these SNPs also appear to modify risk in *BRCA1 *and/or *BRCA2 *mutation carriers [8]. Not all of the breast cancer-associated SNPs assessed have been found to modify risk in carriers, however, and some of the risk associations are specific for *BRCA2 *mutation carriers only and not *BRCA1 *[8]. While GWA studies specifically addressing risk for *BRCA1 *and/or *BRCA2 *carriers are a more direct approach to identifying modifiers of these genes using an agnostic approach, GWA studies require large sample sizes to identify genetic modifiers with confidence. To address the problem of inadequate sample size, the CIMBA was established in 2005 to link clinical and epidemiological data from many groups from around the world [17]. The GWA approach is still limited, however, in that study designs involve predefined stringent selection criteria for which SNPs identified from the initial whole genome scan are going to be analysed in subsequent replication studies, a study design enforced by current genotyping costs. Moreover, GWA studies are often limited in information about exogenous risk factors, such as environmental exposures, which confounds any effort to explore the effect of environmental factors in modifying gene-disease associations. Global gene expression analysis as a means to agnostically identify candidate genetic modifiers has the potential to prioritise SNPs for candidate genes for association studies. This may be particularly valuable given recent observations that SNPs associated with risk of cancer in the general population appear to reside in noncoding regions that may modulate gene expression.

An alternative approach to prioritising SNPs and candidate genes for association studies in *BRCA1 *and *BRCA2 *mutation carriers could rely on the selection of genes displaying associations with *BRCA1 *or *BRCA2 *mutation status at the expression level in response to DNA damage. In a previous study, we used a novel combinatorial approach to identify a subset of 20 irradiation responsive genes as high-priority candidate *BRCA1 *and/or *BRCA2 *modifier genes [18]. The expression levels of these genes were shown to be associated with *BRCA1 *and/or *BRCA2 *mutation status in irradiated lymphoblastoid cell lines from female carriers when compared against irradiated lymphoblastoid cell lines from healthy controls. Furthermore, each of the genes were tagged with one or more SNPs shown to be associated with breast cancer risk from the Cancer Genetic Markers of Susceptibility (CGEMS) Phase 1 Breast Cancer Whole Genome Association Scan [19,20]. In the present study we investigated the association of these polymorphisms, tagged to genes demonstrated *in vitro *to be involved in irradiation response, with risk of breast cancer for *BRCA1 *and *BRCA2 *mutation carriers.

## Materials and methods

### Study participants

Eligibility of study participants was restricted to female *BRCA1 *or *BRCA2 *pathogenic mutation carriers who were aged 18 years or older. Fifteen clinic and population-based research studies from the USA, Canada, Australia, the UK and Europe submitted data to the present study (Table 1). Information collected included year of birth, age at diagnosis of breast cancer or ovarian cancer, age at last observation, family membership, ethnicity and information on bilateral prophylactic mastectomy and oophorectomy. All centres have obtained informed consent from study participants and the institutional review board approved protocols. In total, this study included up to 4,724 *BRCA1 *and 2,693 *BRCA2 *eligible female mutation carriers. Of the 2,193 and 1,189 unaffected *BRCA1 *and *BRCA2 *carriers, respectively, 972 (44.3%) and 589 (49.5%) had a relative that was in the affected group.

**Table 1:** Distribution of BRCA1 and BRCA2 mutation carriers by study site

Study	Country^a^	*BRCA1*	*BRCA2*	Genotyping platform
HEBON	The Netherlands	807	308	iPLEX^b^; Golden Gate^c^
EMBRACE	UK	841	656	iPLEX^b^; Golden Gate^c^
FCCC	USA	82	53	iPLEX^b^; Golden Gate^c^
GC-HBOC	Germany	398	163	Golden Gate^c^
GEMO	France/USA	408	226	Golden Gate^c^
Georgetown	USA	27	14	iPLEX^b^; Golden Gate^c^
HEBCS	Finland	103	104	iPLEX^b^; Golden Gate^c^
ILUH	Iceland	6	87	iPLEX^b^; Golden Gate^c^
kConFab	Australia/New Zealand	531	427	iPLEX^b^; Golden Gate^c^
Mayo	USA	227	123	iPLEX^b^; Golden Gate^c^
ModSQuaD	USA	158	91	Golden Gate^c^
MUV	Austria	298	126	iPLEX^b^; Golden Gate^c^
PBCS	Italy	76	43	iPLEX^b^
SWE-BRCA	Sweden	489	141	iPLEX^b^; Golden Gate^c^
UPENN	USA	273	131	iPLEX^b^; Golden Gate^c^

EMBRACE, Epidemiological Study of BRCA1 and BRCA2 Mutation Carriers; FCCC, Fox Chase Cancer Center; HEBON, Hereditary Breast and Ovarian Cancer Research Group Netherlands; ILUH, Iceland Landspitali - University Hospital Study; kConFab, Kathleen Cunningham Consortium for Research into Familial Breast Cancer; ModSQuaD, Modifier Study of Quantitative Effects on Disease; MUV, Medical University of Vienna; PBCS, Pisa Breast Cancer Study. ^a^Coordinating centre. ^b^Samples were genotyped at the Queensland Institute of Medical Research. ^c^Samples were genotyped at the Mayo Clinic.

### SNP selection and genotyping

In a previous report, we proposed 13 genes (*ARHGEF2*, *HNRPDL*, *IL4R*, *JUND*, *LSM2*, *MAGED2*, *MLF2*, *MS4A1*, *SMAD3*, *STIP1*, *THEM2*, *TOMM40*, *VNN2*) as candidate modifiers of breast cancer risk for *BRCA1 *mutation carriers, and 14 genes (*ARHGEF2*, *JUND*, *MLF2*, *SMAD3*, *STIP1*, *THEM2*, *TOMM40*, *ABL1*, *ELMO1*, *EPM2AIP1*, *PER1*, *PLCG2*, *PLD3*, *SLC20A1*) as candidate modifiers of breast cancer risk for *BRCA2 *mutation carriers (see Additional file 1) [18]. Thirty-seven SNPs denoted by CGEMS as being tagged to these genes were initially identified as showing some association with breast cancer risk (*P *< 0.05) (see Additional file 2). Of these 37 SNPs, a panel of 32 variants were selected after successful assay design and genotyped on two platforms, using the Illumina GoldenGate assay (Illumina Inc., San Diego, California, USA) and the Sequenom MassARRAY iPLEX platform (Sequenom, San Diego, CA, USA), as previously described [21,22]. The genotyping method used for each participating study is detailed in Table 1. Five SNPs tagged to five candidate genes (*JUND*, *MAGED2*, *MLF2*, *MLH1*, *STIP1*) had call rates <95% and were excluded from the analysis. The minor allele frequencies of three SNPs (rs2893535 - *ELMO1*, minor allele frequency = 0.033; rs2304911 - *PER1*, minor allele frequency = 0.043; and rs3802957 - *MS4A1*, minor allele frequency = 0.04) were considered too small for reliable analysis. The number of genes assessed for their associations with breast cancer risk for *BRCA1 *and *BRCA2 *mutation carriers was therefore eight and 10, respectively.

### Statistical methods

Relative risks (RRs) and 95% confidence intervals were estimated using weighted Cox proportional hazards models. Each subject was followed from birth to the earliest of breast cancer, bilateral mastectomy, ovarian cancer, last follow-up, or age 80. The phenotype of interest was time to breast cancer. Mutation-specific weights were calculated using the age distribution of affected and unaffected individuals according to the methods previously outlined by Antoniou and colleagues [23]. Analyses were stratified by year of birth, ethnicity, country of residence, study site, and mutation status. A robust variance estimate was used to account for relatedness amongst individuals. Primary SNP analyses assumed a log-additive relationship between the number of minor alleles carried by each individual and time to breast cancer. Wald *P *values below 0.05 were declared of interest. Secondary analyses were carried out in which RR estimates were separately generated for those carrying one and two copies of the minor allele versus those with two copies of the major allele. Between-study heterogeneity was examined in each SNP by including an interaction term between the genotype and study centre.

Owing to the highly-selected nature of subjects, a number of sensitivity analyses were examined. To limit the effect of potential survival bias, subjects diagnosed more than 5 years prior to study enrolment were excluded (number affected analysed = 1,342 and 762 for *BRCA1 *and *BRCA2 *carriers, respectively). Other models were examined that excluded women with ovarian cancer (number excluded = 491 and 151 *BRCA1 *and *BRCA2 *carriers, respectively). Finally, as risk of breast cancer is reduced after bilateral oophorectomy [24,25], analyses were carried out treating oophorectomy as a time-dependent covariate in the Cox proportional hazards models. All *P *values are two sided and analyses were carried out using R software [26].

## Results and Discussion

A cohort of up to 4,724 *BRCA1 *and 2,693 *BRCA2 *female mutation carriers was used for the present study. Of these, 4,035 mutation carriers were diagnosed with breast cancer or ovarian cancer at the end of follow-up and 3,382 were censored as unaffected at a mean age of 44 years. The patient characteristics of *BRCA1 *and *BRCA2 *mutation carriers are presented in Table 2.

**Table 2:** Patient characteristics

Characteristic	*BRCA1 *mutation carriers		*BRCA2 *mutation carriers	
		
	Unaffected	Breast cancer	Unaffected	Breast cancer
Number of carriers	2,193	2,531	1,189	1,504
Length of follow-up (person-years)	93,521	102,870	53,147	66,764
Mean (SD) age at censure (years)	43 (12.6)	41 (9.4)	45 (13.2)	44 (9.7)
Age at censure, *n *(%)				
< 30 years	344 (16%)	252 (10%)	144 (12%)	52 (3%)
30 to 39 years	658 (30%)	1060 (42%)	343 (29%)	474 (32%)
40 to 49 years	608 (28%)	809 (32%)	331 (28%)	587 (39%)
50 to 59 years	374 (17%)	310 (12%)	215 (18%)	273 (18%)
60 to 69 years	143 (6%)	87 (3%)	101 (8%)	93 (6%)
70+ years	66 (3%)	13 (1%)	55 (5%)	25 (2%)
Year of birth, *n *(%)				
Before 1949	523 (24%)	840 (33%)	281 (24%)	602 (40%)
1949 to 1959	508 (23%)	816 (32%)	307 (26%)	518 (35%)
1960 to 1968	594 (27%)	602 (24%)	302 (25%)	303 (20%)
After 1968	568 (26%)	273 (11%)	299 (25%)	81 (5%)
Oophorectomy	260 (12%)	77 (3%)	126 (11%)	47 (3%)
Ethnicity, *n *(%)				
Caucasian	2127 (97%)	2446 (97%)	1159 (97%)	1464 (97%)
Ashkenazi Jewish	66 (3%)	85 (3%)	30 (3%)	40 (3%)

SD, standard deviation.

The RR estimates for the association between SNP genotypes and risk of breast cancer for *BRCA1 *and *BRCA2 *mutation carriers are presented in Table 3 and Table 4 respectively. Of the 24 SNPs that passed quality control, the minor alleles of two SNPs were found to be associated with increased risk for *BRCA1 *mutation carriers (rs10242920 - *ELMO1*, *P *= 0.043; and rs480092 - *LSM2*, *P *= 0.015) and the minor alleles of three SNPs to be associated with increased risk for *BRCA2 *mutation carriers (rs1559949 - *HNRPDL*, *P *= 0.021; rs3825977 - *SMAD3*, *P *= 0.018; and rs7166081 - *SMAD3*, *P *= 0.004). The minor alleles of two SNPs, rs1559949 (*HNRPDL*) and rs3808814 (*ABL1*), were associated with decreased risk for *BRCA1 *(*P *= 0.022) and *BRCA2 *(*P *= 0.030) mutation carriers, respectively.

**Table 3:** Genotype distributions of 24 candidate modifier SNPs and hazard ratio estimates for BRCA1 mutation carriers

SNP	Gene	Minor allele	MAF	Heterozygous		Homozygous		Per allele		*P*_trend_
							
				HR	95% CI	HR	95% CI	HR	95% CI	
rs7026988	*ABL1*	A	0.12	1.08	0.88 to 1.34	1.61	0.82 to 3.15	1.13	0.93 to 1.36	0.212
rs3808814	*ABL1*	A	0.09	0.90	0.72 to 1.14	0.65	0.22 to 1.91	0.89	0.72 to 1.10	0.284
rs1889532	*ARHGEF2*	G	0.25	0.99	0.83 to 1.18	1.12	0.80 to 1.56	1.03	0.90 to 1.18	0.708
rs10242920	*ELMO1*	A	0.24	1.06	0.89 to 1.27	1.61	1.12 to 2.32	1.16	1.00 to 1.33	*0.043*
rs6964474	*ELMO1*	C	0.22	1.08	0.90 to 1.29	0.71	0.49 to 1.02	0.96	0.84 to 1.10	0.568
rs2541095	*ELMO1*	G	0.12	1.03	0.84 to 1.27	1.12	0.48 to 2.63	1.04	0.86 to 1.26	0.683
rs6956864	*ELMO1*	G	0.41	1.04	0.76 to 1.42	1.35	0.18 to 10.03	1.05	0.78 to 1.42	0.755
rs1559949	*HNRPDL*	G	0.14	0.78	0.65 to 0.94	0.91	0.51 to 1.64	0.82	0.70 to 0.97	*0.022*
rs4285076	*HNRPDL*	A	0.29	0.95	0.80 to 1.12	1.00	0.73 to 1.37	0.98	0.86 to 1.11	0.746
rs4787956	*IL4R*	G	0.34	0.99	0.83 to 1.18	1.11	0.84 to 1.46	1.03	0.91 to 1.17	0.611
rs16976728	*IL4R*	A	0.38	0.92	0.77 to 1.10	1.07	0.82 to 1.39	1.00	0.88 to 1.13	0.978
rs480092	*LSM2*	G	0.16	1.25	1.04 to 1.51	1.30	0.81 to 2.08	1.21	1.04 to 1.42	*0.015*
rs2253820	*PER1*	A	0.17	1.02	0.9 to 1.16	0.72	0.52 to 1.00	0.96	0.87 to 1.06	0.412
rs4888201	*PLCG2*	A	0.16	1.10	0.91 to 1.34	1.39	0.77 to 2.51	1.13	0.95 to 1.33	0.168
rs10514519	*PLCG2*	A	0.18	1.06	0.88 to 1.28	1.59	0.92 to 2.75	1.11	0.95 to 1.31	0.195
rs4997772	*PLCG2*	A	0.39	1.13	0.95 to 1.35	1.07	0.84 to 1.37	1.05	0.94 to 1.18	0.377
rs3936112	*PLCG2*	A	0.39	0.98	0.87 to 1.10	0.97	0.82 to 1.16	0.98	0.91 to 1.07	0.700
rs4254419	*PLD3*	A	0.15	0.98	0.81 to 1.18	0.87	0.50 to 1.52	0.96	0.82 to 1.13	0.648
rs10758	*SLC20A1*	G	0.26	0.98	0.82 to 1.17	0.95	0.68 to 1.33	0.98	0.85 to 1.12	0.729
rs3825977	*SMAD3*	A	0.20	0.98	0.88 to 1.11	0.94	0.71 to 1.24	0.98	0.89 to 1.07	0.638
rs7166081	*SMAD3*	G	0.24	0.98	0.87 to 1.11	1.03	0.80 to 1.33	1.00	0.91 to 1.10	0.995
rs3777663	*THEM2*	G	0.24	1.02	0.86 to 1.22	1.17	0.84 to 1.63	1.05	0.92 to 1.20	0.453
rs2075642	*TOMM40*	A	0.20	0.97	0.81 to 1.16	1.06	0.69 to 1.63	0.99	0.86 to 1.15	0.931
rs12211125	*VNN2/VNN3*	G	0.09	0.96	0.83 to 1.11	1.19	0.73 to 1.94	0.99	0.87 to 1.12	0.882

MAF, minor allele frequency; HR, hazard ratio; CI, confidence interval.

**Table 4:** Genotype distributions of 24 candidate modifier SNPs and hazard ratio estimates for BRCA2 mutation carriers

SNP	Gene	Minor allele	MAF	Heterozygous		Homozygous		Per allele		*P*_trend_
							
				HR	95% CI	HR	95% CI	HR	95% CI	
rs7026988	*ABL1*	A	0.12	0.96	0.73 to 1.26	0.93	0.41 to 2.10	0.96	0.76 to 1.20	0.713
rs3808814	*ABL1*	A	0.09	0.72	0.51 to 1.02	0.50	0.17 to 1.42	0.71	0.53 to 0.97	*0.030*
rs1889532	*ARHGEF2*	G	0.25	1.32	1.05 to 1.67	0.99	0.64 to 1.54	1.13	0.95 to 1.33	0.172
rs10242920	*ELMO1*	A	0.24	1.01	0.79 to 1.30	0.86	0.52 to 1.43	0.97	0.80 to 1.17	0.747
rs6964474	*ELMO1*	C	0.22	1.09	0.86 to 1.40	1.47	0.89 to 2.45	1.15	0.95 to 1.39	0.153
rs2541095	*ELMO1*	G	0.12	1.04	0.8 to 1.35	0.72	0.27 to 1.94	1.00	0.78 to 1.26	0.971
rs6956864	*ELMO1*	G	0.41	0.76	0.48 to 1.18	2.20	0.16 to 29.7	0.78	0.50 to 1.22	0.279
rs1559949	*HNRPDL*	G	0.14	1.29	0.96 to 1.72	2.06	0.99 to 4.28	1.33	1.04 to 1.70	*0.021*
rs4285076	*HNRPDL*	A	0.29	0.86	0.67 to 1.09	1.47	0.95 to 2.26	1.03	0.85 to 1.25	0.737
rs4787956	*IL4R*	G	0.34	1.10	0.87 to 1.41	1.31	0.90 to 1.91	1.13	0.95 to 1.35	0.167
rs16976728	*IL4R*	A	0.38	1.16	0.91 to 1.48	1.38	0.95 to 1.99	1.17	0.98 to 1.39	0.075
rs480092	*LSM2*	G	0.16	0.92	0.72 to 1.18	1.09	0.55 to 2.16	0.96	0.78 to 1.19	0.735
rs2253820	*PER1*	A	0.17	0.85	0.70 to 1.02	1.13	0.68 to 1.87	0.90	0.77 to 1.06	0.209
rs4888201	*PLCG2*	A	0.16	0.98	0.75 to 1.27	1.16	0.54 to 2.52	1.01	0.80 to 1.27	0.964
rs10514519	*PLCG2*	A	0.18	0.85	0.66 to 1.09	1.92	0.95 to 3.88	0.99	0.79 to 1.24	0.933
rs4997772	*PLCG2*	A	0.39	1.21	0.95 to 1.55	1.26	0.89 to 1.78	1.14	0.97 to 1.34	0.107
rs3936112	*PLCG2*	A	0.39	0.91	0.76 to 1.09	0.94	0.73 to 1.22	0.96	0.85 to 1.08	0.483
rs4254419	*PLD3*	A	0.15	0.95	0.73 to 1.22	0.74	0.36 to 1.51	0.92	0.74 to 1.14	0.448
rs10758	*SLC20A1*	G	0.26	1.12	0.88 to 1.42	1.11	0.67 to 1.84	1.08	0.90 to 1.30	0.388
rs3825977	*SMAD3*	A	0.20	1.10	0.91 to 1.33	1.83	1.23 to 2.73	1.20	1.03 to 1.40	*0.018*
rs7166081	*SMAD3*	G	0.24	1.17	0.97 to 1.42	1.74	1.21 to 2.49	1.25	1.07 to 1.45	*0.004*
rs3777663	*THEM2*	G	0.24	0.95	0.75 to 1.21	1.11	0.69 to 1.79	0.99	0.82 to 1.20	0.945
rs2075642	*TOMM40*	A	0.20	1.14	0.89 to 1.46	1.19	0.68 to 2.09	1.12	0.92 to 1.37	0.267
rs12211125	*VNN2 /VNN3*	G	0.09	1.01	0.81 to 1.26	1.31	0.49 to 3.56	1.02	0.83 to 1.26	0.818

MAF, minor allele frequency; HR, hazard ratio; CI, confidence interval.

**Figure 1:**
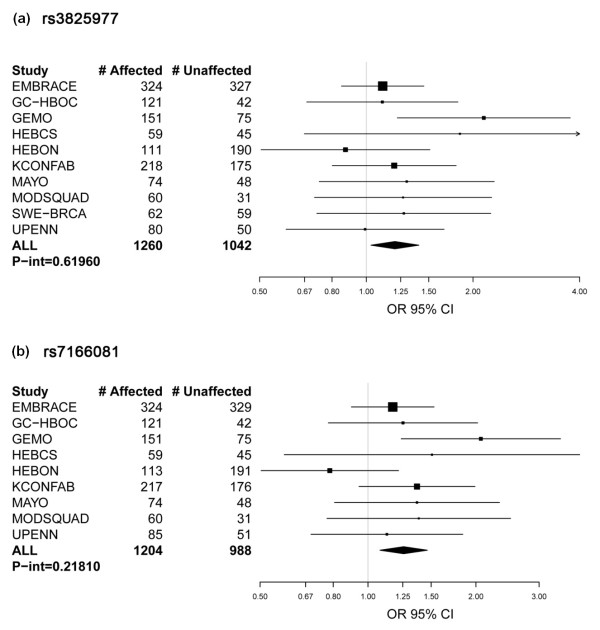
***BRCA2 *plot of relative risk for rs3825977 and rs7166081 at the *SMAD3 *locus**. *BRCA2 *plots of study group-specific relative risk (RR) for rs3825977 and rs7166081 at the *SMAD3 *locus. Study groups with 70 or more carriers and tests of heterogeneity are shown for **(a) **rs3825977 (overall RR (95% confidence interval (CI)) = 1.20 (1.03, 1.40), *P*_trend _= 0.018) and **(b) **rs7166081 (overall RR (95% CI) = 1.25 (1.07, 1.45), *P*_trend _= 0.004). OR, odds ratio.

All SNPs selected for the present study (see Additional file 2) had previously been reported to be at least marginally associated (*P *< 0.05) with breast cancer risk through the CGEMS Phase 1 Breast Cancer Whole Genome Association Scan [18], and to be tagged to a gene whose expression level was associated with *BRCA1 *and/or *BRCA2 *mutation status in irradiated lymphoblastoid cell lines [18]. The minor allele of four out of six SNPs shown here to be associated with risk in *BRCA1 *mutation carriers (rs1559949 - *HNRPDL*; rs480092 - *LSM2*) or *BRCA2 *(rs3825977 and rs7166081 - *SMAD3*) had risk estimates for the homozygous genotype that were concordant with the odds ratio reported by the CGEMS study (Table 3 and 4, and Additional file 2). Furthermore, the expression of *HNRPDL *and *LSM2 *was associated with *BRCA1 *mutation status and the expression of *SMAD3 *was associated with BRCA2 mutation status [18]. The risk estimate of rs10242920 (*ELMO1*) was also concordant with the odds ratio determined by the CGEMS study; and although the expression of *ELMO1 *was not associated with *BRCA1 *mutation status at *P *< 0.001, there was an association with gene expression at *P *< 0.005 [18]. In contrast, the risk estimates of rs3808814 (*ABL1*) and rs1559949 (*HNRPDL*) in *BRCA2 *mutation carriers are not concordant with the odds ratio determined by the CGEMS study. Forest plots of study groups with 70 or more carriers and tests of heterogeneity are shown for two of the most significant SNPs (rs3825977, *P*-het = 0.619 and rs7166081, *P*-het = 0.218 at the *SMAD3 *locus), stratified by study site (Figure 1). The minor alleles of rs3825977 and rs7166081 are in high linkage disequilibrium (*r*^2 ^= 0.77), which would be expected if their association with increased breast cancer risk is *bona fide*.

Although further study is required to confirm whether genetic variation in *SMAD3 *plays a role in modifying risk of breast cancer, SMAD3 has been shown to interact with the BRCA2 protein - suggesting a possible mechanism through which SMAD3 may modify BRCA2 function [27]. Furthermore, SMAD3 is a critical regulatory factor of the transforming growth factor beta pathway, which is known to play a key role in the development of breast cancer as well as many other cancers [28,29]. In addition, a recent study comparing dense breast tissue (a known breast cancer risk factor) with nondense tissue identified reduced expression of SMAD3 to be associated with dense tissue, indirectly supporting a role of SMAD3 expression with breast cancer risk [29].

Choosing candidate *BRCA1 *and *BRCA2 *modifier genes from a novel combinatorial approach [18], we show that four SNPs tagged to three of the 14 candidate genes show an association with breast cancer risk for *BRCA1 *or *BRCA2 *mutation carriers. We initiated the present study, however, with the expectation that SNPs in eight of the 14 genes may be associated with altered expression in *BRCA1 *mutation carriers, and 10 of the 14 genes with altered expression in *BRCA2 *carriers. We can thus argue that three out of 18 (17%) valid comparisons showed an association with risk. For either interpretation, the rate of observed association is greater than the one in 20 (5%) expected by chance. In addition, *post hoc *mining of the expression dataset showed that another SNP (rs10242920 - *ELMO1*) association that was consistent with the effect reported in the CGEMS dataset was also actually associated with altered expression in carriers, albeit with less significance (*P *= 0.005) than originally used for gene and SNP selection. These findings suggest that the combinatorial approach may be a useful method to prioritise candidate modifier genes for polymorphism association studies. It is notable that CIMBA GWA studies of *BRCA1 *and *BRCA2 *mutation carriers are currently underway [30]. One might therefore anticipate that the combinatorial approach would provide even greater enrichment for prioritising SNPs from GWA studies that directly relate to the disease state under study. Further studies with larger cohort size are therefore warranted to assess the benefit of carrying out such an approach.

## Conclusions

We have explored the value of using biological information embedded in gene expression data to prioritise candidate modifier genes for SNP association studies. Using this combinatorial approach we were able to demonstrate a threefold enrichment of genes that contain SNPs associated with breast cancer risk for *BRCA1 *or *BRCA2 *mutation carriers. Most notable was the evidence that the *SMAD3 *gene, which encodes a key regulatory protein in the transforming growth factor beta signalling pathway, may contribute to increased risk of breast cancer in *BRCA2 *mutation carriers. These results suggest that the combinatorial approach may be a useful method to prioritise candidate modifier genes for polymorphism association studies.

## Abbreviations

CGEMS: Cancer Genetic Markers of Susceptibility; CIMBA: Consortium of Investigators of Modifiers of *BRCA1 *and *BRCA2*; GWA: genome-wide association; RR: relative risk; SNP: single nucleotide polymorphism.

## Competing interests

The authors declare that they have no competing interests.

## Authors' contributions

LCW, ABS and FJC conceived and designed the study. LCW, GC-T, ABS and FJC coordinated the study, and LCW drafted the manuscript. ABS and FJC supervised the analysis and participated in manuscript writing. ZSF and VSP carried out the statistical analysis, and ZSF contributed to the manuscript writing. XW, RT, NML, JB, and XC processed samples and acquired data. DS-L, CT, SG, SM, DM, J-PF, CD, RKS, BW, CE, IS, HD, AM, FBH, MV, MJH, AMWvdO, MRN, MGEMA, CMA, CJvA, PD, MMG, QW, CIS, DFE, SP, MC, CTO, DF, PH, DGE, FL, RE, LI, CC, RD, DE, K-RO, JC, TR, KLN, SMD, CFS, DG-K, A-CD, GP, AKG, TH, HN, BAA, MAC, HO, UK, AL, BA, PK, BM, OMS, LM, ACA, GC-T and FJC provided samples and information on the *BRCA1 *and *BRCA2 *mutation carriers included in this study. SH and OMS provided assistance with mutation nomenclature and classifications. LM and ACA maintained the database of *BRCA1 *and *BRCA2 *mutation carriers. All authors read and approved the manuscript.

## Supplementary Material

Additional file 1 **Supplementary Table S1. Genes predicted to modify risk**. Genes predicted to modify risk in *BRCA1 *and/or *BRCA2 *mutation carriers by Walker and colleagues [18]. Click here for file

Additional file 2 **Supplementary Table S2. List of 37 candidate *BRCA1/2 *risk modifier SNPs**. Each SNP listed was tagged to a gene and shown to be associated with breast cancer risk from the CGEMS Study version 1. Click here for file
