# Supplementary material for: Evidence for SMAD3 as a modifier of breast cancer risk in BRCA2 mutation carriers
Source: Breast Cancer Res. 2010 Nov 29;12(6):R102. doi: 10.1186/bcr2785 (PMC3046447; doi:10.1186/bcr2785)
Supplement: Additional file 1 — Supplementary Table S1. Genes predicted to modify risk. Genes predicted to modify risk in BRCA1 and/or BRCA2 mutation carriers by Walker and colleagues [18]. [file bcr2785-S1.DOCX]

**Supplementary Table 1.** Genes predicted to modify risk in *BRCA1* and/or *BRCA2* mutation carriers by Walker *et al* [18]

| **Gene symbol** | **Gene name** | ***BRCA1*** | ***BRCA2*** |
| --- | --- | --- | --- |
| *ARHGEF2* | rho/rac guanine nucleotide exchange factor (GEF) 2 | Yes | Yes |
| *HNRPDL* | heterogeneous nuclear ribonucleoprotein D-like | Yes |  |
| *IL4R* | interleukin 4 receptor | Yes |  |
| *JUND* | jun D proto-oncogene | Yes | Yes |
| *LSM2* | LSM2 homolog, U6 small nuclear RNA associated (S. cerevisiae) | Yes |  |
| *MAGED2* | melanoma antigen family D, 2 | Yes |  |
| *MLF2* | myeloid leukemia factor 2 | Yes | Yes |
| *MS4A1* | membrane-spanning 4-domains, subfamily A, member 1 | Yes |  |
| *SMAD3* | SMAD family member 3 | Yes | Yes |
| *STIP1* | stress-induced-phosphoprotein 1 | Yes | Yes |
| *THEM2* | thioesterase superfamily member 2 | Yes | Yes |
| *TOMM40* | translocase of outer mitochondrial membrane 40 homolog (yeast) | Yes | Yes |
| *VNN2* | vanin 2 | Yes |  |
| *ABL1* | v-abl Abelson murine leukemia viral oncogene homolog 1 |  | Yes |
| *ELMO1* | engulfment and cell motility 1 |  | Yes |
| *EPM2AIP1* | EPM2A (laforin) interacting protein 1 |  | Yes |
| *PER1* | period homolog 1 (Drosophila) |  | Yes |
| *PLCG2* | phospholipase C, gamma 2 (phosphatidylinositol-specific) |  | Yes |
| *PLD3* | phospholipase D family, member 3 |  | Yes |
| *SLC20A1* | solute carrier family 20 (phosphate transporter), member 1 |  | Yes |
