# Supplementary material for: Evidence for SMAD3 as a modifier of breast cancer risk in BRCA2 mutation carriers
Source: Breast Cancer Res. 2010 Nov 29;12(6):R102. doi: 10.1186/bcr2785 (PMC3046447; doi:10.1186/bcr2785)
Supplement: Additional file 2 — Supplementary Table S2. List of 37 candidate BRCA1/2 risk modifier SNPs. Each SNP listed was tagged to a gene and shown to be associated with breast cancer risk from the CGEMS Study version 1. [file bcr2785-S2.DOCX]

**Supplementary Table 2.** List of 37 candidate *BRCA1/2* risk modifier SNPs

| **SNP** | **Gene** | **Chr** | **CGEMS Study Results** | | |
| --- | --- | --- | --- | --- | --- |
|  |  |  | **p-value^a^, version 1.0 (p-value, version 2.0)** | **Heterozygous OR** | **Homozygous OR** |
| rs7026988 | *ABL1* | 9 | 0.0320 (0.0298) | 1.2117 | 1.9362 |
| rs3808814 | *ABL1* | 9 | 0.0127 (0.0139) | 1.0636 | 6.8457 |
| rs3824400 | *ABL1* | 9 | 0.0411 (0.0386) | 1.2073 | 1.9187 |
| rs1889532 | *ARHGEF2* | 1 | 0.0320 (0.0326) | 1.1782 | 1.4713 |
| rs10242920 | *ELMO1* | 7 | 0.0209 (0.0224) | 0.7987 | 1.1365 |
| rs2893535 | *ELMO1* | 7 | 0.0318 (ND) | 1.4359 | ND |
| rs6964474 | *ELMO1* | 7 | 0.0486 (0.0455) | 1.0774 | 0.6444 |
| rs2541095 | *ELMO1* | 7 | 0.0158 (0.0153) | 1.3292 | 1.1601 |
| rs6956864 | *ELMO1* | 7 | 0.0292 (ND) | 1.4382 | ND |
| rs2041854 | *ELMO1* | 7 | 0.0313 (ND) | 1.5137 | ND |
| rs17171046 | *ELMO1* | 7 | 0.0420 (0.0427) | 0.9342 | 0.4411 |
| rs4548075 | *ELMO1* | 7 | 0.0124 (0.0126) | 1.2947 | 1.2985 |
| rs1559949 | *HNRPDL* | 4 | 0.0165 (0.0169) | 0.7556 | 0.7584 |
| rs4285076 | *HNRPDL* | 4 | 0.0380 (0.0390) | 1.1283 | 1.4503 |
| rs4787956 | *IL4R* | 16 | 0.0225 (0.0224) | 0.963 | 0.6973 |
| rs16976728 | *IL4R* | 16 | 0.0217 (0.0210) | 0.9333 | 0.7063 |
| rs2384992 | *JUND* | 19 | 0.0194 (0.0197) | 1.1238 | 0.6856 |
| rs480092 | *LSM2* | 6 | 0.0465 (0.0475) | 0.7964 | 1.1435 |
| rs11555927 | *MAGED2* | X | 0.0236 (0.0250) | 0.8228 | 0.5683 |
| rs2302371 | *MLF2* | 12 | 0.0494 (0.0484) | 1.234 | 1.6035 |
| rs1799977 | *MLH1* | 3 | 0.0354 (0.0350) | 0.9338 | 0.6748 |
| rs3802957 | *MS4A1* | 11 | 0.0092 (ND) | 0.6732 | ND |
| rs2253820 | *PER1* | 17 | 0.0037 (0.0040) | 0.9647 | 0.4071 |
| rs2304911 | *PER1* | 17 | 0.0175 (ND) | 0.7003 | ND |
| rs4888201 | *PLCG2* | 16 | 0.0390 (0.0383) | 0.7937 | 1.0482 |
| rs10514519 | *PLCG2* | 16 | 0.0409 (0.0397) | 0.81 | 0.7339 |
| rs4997772 | *PLCG2* | 16 | 0.0433 (0.0426) | 1.2548 | 1.1841 |
| rs3936112 | *PLCG2* | 16 | 0.0082 (0.0084) | 1.1121 | 1.5108 |
| rs4254419 | *PLD3* | 19 | 0.0173 (0.0170) | 1.2863 | 0.727 |
| rs10758 | *SLC20A1* | 2 | 0.0259 (0.0251) | 0.8013 | 1.0954 |
| rs3825977 | *SMAD3* | 15 | 0.0117 (0.0117) | 0.77 | 1.1359 |
| rs7166081 | *SMAD3  / AAGAB* | 15 | 0.0016 (0.0016) | 0.7563 | 1.2644 |
| rs3751122 | *STIP1* | 11 | 0.0282 (0.0754) | 0.7751 | ND |
| rs3777663 | *THEM2* | 6 | 0.0130 (0.0138) | 0.7737 | 1.0474 |
| rs9356943 | *THEM2* | 6 | 0.0290 (0.0297) | 1.2095 | 1.3694 |
| rs2075642 | *TOMM40* | 19 | 0.0159 (0.0160) | 1.2973 | 1.0536 |
| rs12211125 | *VNN2 / VNN3* | 6 | 0.0103 (0.0120) | 1.2608 | 3.1503 |

**^a^ Adjusted score (http://cgems.cancer.gov/)**

**Abbreviations: SNP, single nucleotide polymorphism; Chr, chromosome, CGEMS, Cancer Genetic Markers of Susceptibility; OR, odds ratio; ND, no data.**
